# Working Towards the Development and Implementation of Precision Mental Healthcare: An Example

**DOI:** 10.1007/s10488-020-01053-y

**Published:** 2020-07-26

**Authors:** Wolfgang Lutz, Brian Schwartz, Juan Martín Gómez Penedo, Kaitlyn Boyle, Anne-Katharina Deisenhofer

**Affiliations:** Department of Psychology, University of Trier, 54286 Trier, Germany

**Keywords:** Routine outcome monitoring, Personalized and precision mental health, Treatment navigation, Artificial intelligence

## Abstract

Leonard Bickman’s (2020) Festschrift paper in the special issue “The Future of Children’s Mental Health Services” on improving mental health services is an impressive reflection of his career, highlighting his major insights and the development of mental health services research as a whole. Five major difficulties in this field’s current research and practice are attentively delineated: poor diagnostics, measurement problems, disadvantages of randomized controlled trials (RCTs), lack of feedback and personalized treatments. Dr. Bickman recommends possible solutions based on his extensive experience and empirical findings. We agree with his thoughts and illustrate how we, challenged with the same problems, have attempted to develop clinically informed research and evidence-based clinical practice. A comprehensive feedback system that deals with the aforementioned problems is briefly described. It includes pre-treatment recommendations for treatment strategies and an empirically informed dropout prediction based on a variety of data sources. In addition to treatment recommendations, continuous feedback as well as individualized treatment adaptation tools are provided during ongoing therapy. New projects are being implemented to further improve the system by including new data assessment strategies and sources, e.g., ecological momentary assessment (EMA) and automated video analysis.

It is an honor to comment on Leonard Bickman’s (2020) Festschrift paper in the special issue “The Future of Children’s Mental Health Services”. Based on his long and outstanding research career, Dr. Bickman presents an exciting overview of his achievements and offers suggestions for future directions in mental health services research. Critical aspects of previous research are discussed, such as the limits of randomized controlled trials (RCTs) and the problems of previous research to improve health care. Further, interesting new approaches to outcome monitoring and precision mental health based on predictions using new statistical tools from artificial intelligence (AI) are suggested to overcome these limitations. A major strength of the paper is that it delivers a comprehensive overview of current difficulties in mental health research, each of which is thoughtfully delineated. In the following, we discuss the points raised in the paper and present a research program that attempts to tackle several of the described difficulties.

The first targets of critical analysis offered in the paper are arbitrary definitions of diagnoses and their lack of clarity and action-guiding information. AI and machine learning are presented as possible solutions to improve reliability via statistically informed diagnoses. The existing diagnostic heterogeneity may be reduced by more sophisticated approaches to classifying symptoms into diagnostic categories. Furthermore, AI is not only able to provide enhanced methods to determine diagnoses, but can incorporate a greater amount of information than the DSM to make a diagnosis.

This suggested solution is related to the second problem. As Bickman attentively points out, despite fast emerging innovations in data analytical methods, the development of novel measures remains largely disregarded. We agree that the results of our statistical methods rely on the quality of the underlying measures. Comparing our psychometric measures with video data from our research clinic, we have also found that clinical impression and patient self-report do not always match. This indicates the need to further investigate this mismatch and develop additional measures.

In response to this problem, the author provides an impressive compilation of possible solutions that should inspire readers to implement some of these innovations. He recommends assessing real-time data in naturalistic environments with ecological momentary assessments (EMA) instead of relying on artificial laboratory data only. Moreover, psychometric data should be augmented by patients’ psychobiological, psychophysiological, and behavioral data. Technical progress regarding video and audio recordings as well as their automated analyses enables researchers to refocus on this often neglected, but extremely relevant data.

Third, the readers are confronted with the disadvantages of RCTs. The author reflects his commitment to RCTs self-critically. Major limitations of RCTs are denominated: lack of external validity, problems with classic inferential statistics, focus on groups instead of individuals, sample selection, reproducibility problems, and others (Howard et al. 1996). Again, machine learning approaches and AI are a proposed solution, however, the importance of the precise analysis of causality is also highlighted. Applying AI may overcome the problem of low internal validity in observational data, allowing RCTs to be replaced by naturalistic trials and augmented by idiographic research (Bickman 2020).

Fourth, Bickman criticizes the lack of learning through feedback. He states that information on the therapeutic process and progress should be assessed and then fed back to therapists, enabling them to learn from the information. This approach is becoming more and more widespread and it is increasingly seen as crucial to therapist development (see also Bickman 2008; Delgadillo et al. 2018; Lambert et al. 2018). The author presents several approaches to examine and implement feedback into clinical practice and highlights the utility of web- and mobile device-based solutions. Besides the technical hurdles to the development and implementation of feedback, he also points out another crucial aspect: implementation strategies must support therapists to use feedback and facilitate the development of positive attitudes towards feedback (see also Lutz et al. 2015).

The fifth major aspect that is discussed in the manuscript is the lack of empirically-based personalization in psychological treatments. Established treatments are only effective for some patients and it remains unclear, which treatment should be applied for which patient. To improve treatment precision by individually selecting the treatment with the highest probability of response, utilizing AI and testing predictions prospectively instead of relying on retrospective analyses only is suggested. With this point, the author tackles a major limitation of recent literature on prediction as a whole. While prediction models tend to overfit to the data that were used to develop them, AI is able to counter overfitting by, e.g., shrinking estimates and cross-validating them k-fold. Moreover, researchers must show that their prediction models work in independent data (e.g., holdout data). However, testing predictions in independent data is not sufficient. Prediction models should be implemented into clinical practice and their usefulness should be evaluated prospectively with regard to effect sizes, treatment length, and dropout. Unfortuanetly this is not often done yet with limited exceptions (Delgadillo & Lutz, 2020).

We acknowledge Dr. Bickman’s achievement, summarizing the major critical points in mental health research and generating new suggestions for the future. We certainly agree that senior as well as younger colleagues should read this paper and be encouraged to implement the recommended solutions in their own research. This would indeed influence mental health research meaningfully and result in important changes in trial designs, data collection and analysis, and the examination of psychotherapy and mental health services as a whole.

## A Path to Precision Mental Healthcare

Having been confronted with the same problems in our own research, in recent years, we have begun to develop a line of research dedicated to overcoming some of these issues. We are currently conducting several research projects that apply patient-focused feedback research, including outcome monitoring, AI, EMA, and concepts from precision medicine (Deisenhofer et al. 2018; Lutz et al. 2018; 2019; Rubel et al. 2019). More than a decade of our department’s research activity has cumulated in the development of a comprehensive feedback system called the Trier Treatment Navigator (TTN). The system combines outcome tracking, prediction and prescription tools, providing feedback to clinicians and supporting them to apply targeted clinical problem solving strategies when poor treatment response is likely (Lutz et al. 2019). Within a portal accessible to therapists, the navigation system is composed of two modules: one offering therapists a personalized pre-treatment recommendation and one supporting therapists during treatment with adaptive recommendations and clinical problem-solving tools. The system is based on psychometric questionnaires routinely collected every session and is one of the first comprehensive feedback systems to be tested and evaluated prospectively.

**Fig. 1:**
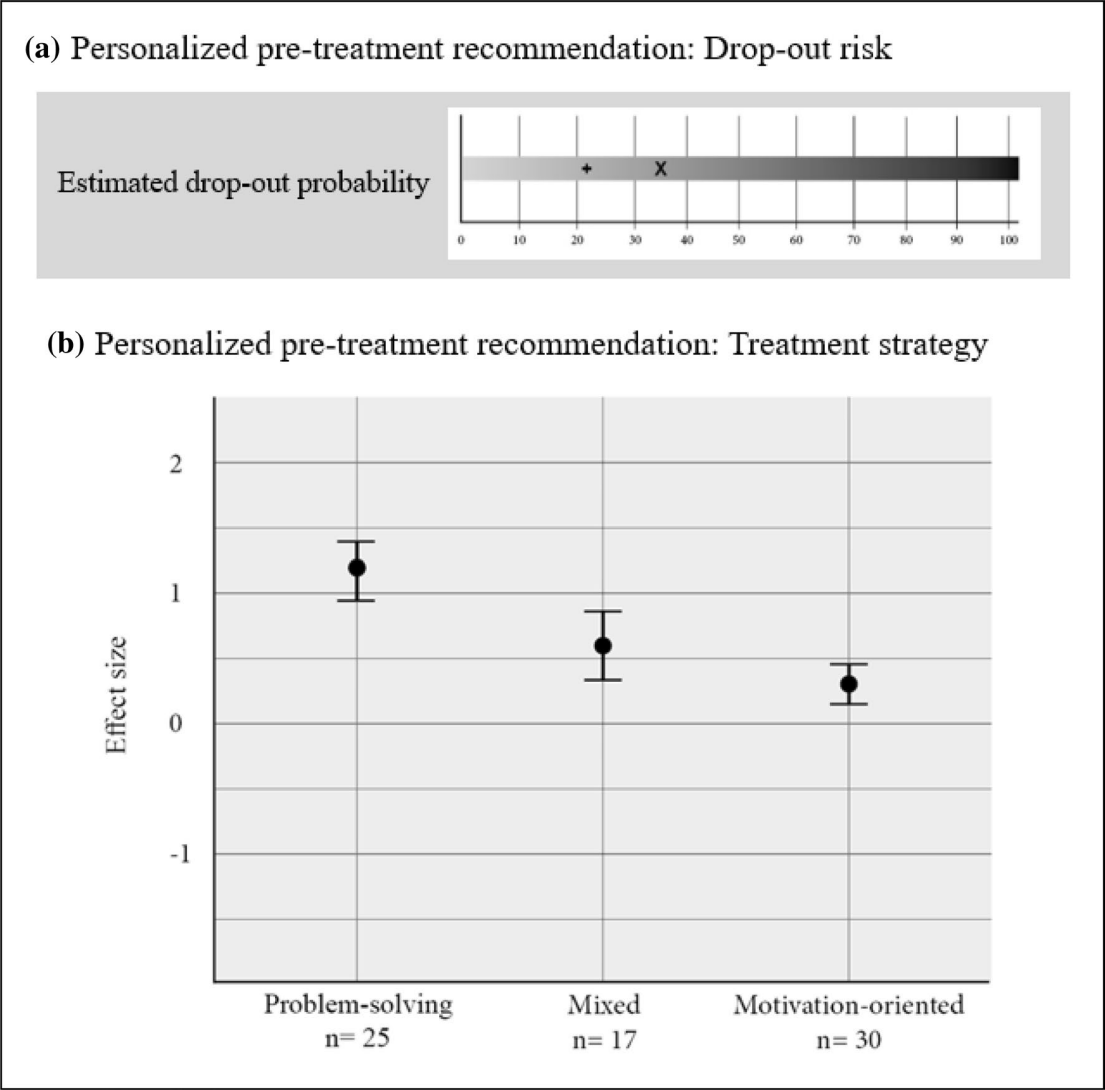
Pre-treatment recommendations as it is displayed to therapists within the navigation system. **a** Drop-out risk (in %): X = patient-specific drop-out probability; +  = average drop-out rate at our outpatient clinic. **b** For a maximum of 30 most similar cases already treated, the average effect size is determined for the three treatment strategies: problem-solving, mixed and motivation-oriented. Problem-solving strategy shows the highest effect size, meaning this strategy was more successful in the cases most similar to this specific patient and is preferable to the other two

Therefore, the standardized diagnostic process as well as personalized predictions for treatment planning can be part of clinical routine. For example, patients in our research clinic are diagnosed using the Structured Clinical Interview for Axis I DSM-IV Disorders-Patient Edition. However, in addition to this limited categorical diagnosis, the TTN provides therapists with empirically-based predictions for each new patient with regard to the probability of dropping out of treatment as well as recommendations for an optimal treatment strategy. Therapists are encouraged to integrate this additional evidence-based information into their treatment plan (see Fig. 1).

Dropout predictions are based on a large sample of already-treated patients (N = 1234). During the system’s development phase, variables predictive of dropout were identified, for example, initial patient severity, personality accentuations, and patients’ level of education. In the system, these variables are used to estimate the specific dropout probability for each patient, which is then fed back to therapists via the portal (Lutz et al. 2019). Additionally, the system provides pre-treatment recommendations with information on the optimal strategy with which to begin treatment. These recommendations are based on the same large sample of already-treated patients. Treatment strategies were developed based on therapist session ratings of the primarily used intervention strategy during the first 10 sessions of treatment (see Fig. 1).

This approach resulted in recommendations of three clinical strategies, defined using session reports: problem-solving, motivation-oriented, and a mix of both strategies. The predictive algorithm was developed using an AI method, the nearest neighbor approach, which has a good relative human-to machine decision-making ratio and is easily clinically comprehensible and communicable. A specific clinical strategy is recommended for an individual patient when, for the most similar cases (nearest neighbors), the average effect size until session 10 is at least 0.1 higher than the averaged effect sizes for both other strategies (see Fig. 1; Lutz et al. 2019).

**Fig. 2:**
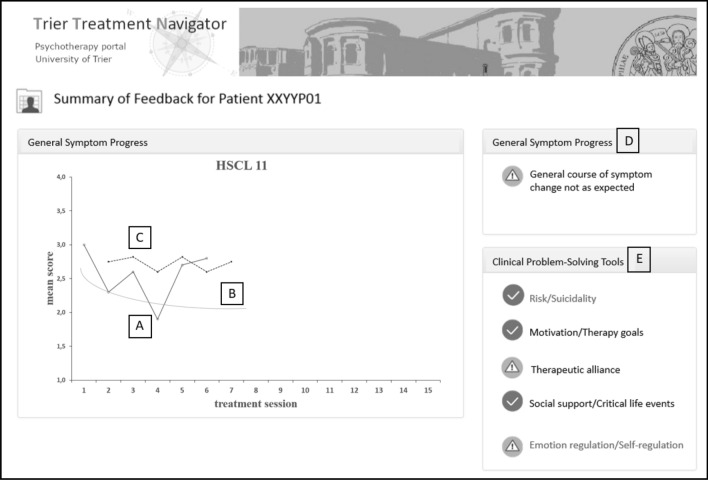
Example screenshot of a patient-specific adaptive treatment recommendation as it is displayed to therapists within the navigation system. **a** Individual patient’s measurement points, measured at the beginning of each session; **b** Expected recovery curve; **c** Failure boundary; **d** As soon as the patient’s score exceeds the failure boundary on the HSCL-11, the therapist receives this warning signal, which is defined in more detail in the clinical problem-solving tools (CPST) below; **e** CPST are divided into five domains. The exclamation mark indicates the domains in which the patient has specific problems. The therapist is able to click on these icons to gain access to the activated tools. The check mark indicates that the patient has few or no problems in this area

The second part of the TTN accompanies therapists during ongoing treatment with adaptive personalized treatment recommendations for patients at risk for treatment failure. With the help of machine learning principles, an individualized expected recovery curve is calculated for each patient. This information is included in the feedback system and presented to therapists alongside the patient’s observed treatment progress (see Fig. 2a, b). Additionally, for each next session, a dynamic failure or risk boundary is presented based on the previous psychometric information compared to the results of already-treated patients (see Fig. 2c). If a patient reports more distress than expected, therapists are prompted with a signal that their patient is off-track and at risk for treatment failure (see Fig. 2d).

For not on track (NOT) cases, therapists are provided with clinical problem-solving tools (CPST; see Lambert et al. 2018). CPSTs are advanced clinical support tools including specific clinical recommendations and supportive therapeutic material to treat patients at risk (including videos, clinical exercises and worksheets), which are presented within the software. They consist of the following problem areas (1) risk/suicidality, (2) motivation/therapy goals, (3) therapeutic alliance, (4) social support and critical life events as well as (5) emotion regulation/self-regulation (see Fig. 2e).

Each problem area or domain is assessed every fifth session by either the Assessment for Signal Clients (ASC; Lambert et al. 2007), the Affective Style Questionnaire (ASQ; Graser et al. 2012) or single items from the Outcome Questionnaire (OQ-30; Ellsworth et al. 2006), and Hopkins Symptom Checklist – Short Form (HSCL-11; Lutz et al. 2006).

The CPST have a shared structure. First, a thematic summary is presented. Second, relevant items answered critically by the patient are displayed. Third, in-depth questions help therapists to reflect on their patients. Fourth, each tool provides recommendations for interventions and strategies that support the therapist to resolve the specific problem. For further details, see Lutz et al. (2019).

As Bickman mentions in his paper, how feedback is implemented into clinical routine is critical. Several studies support the idea that therapists attitudes towards and usage of feedback are important moderators of feedback success (de Jong et al. 2012; Lutz et al. 2015). Being aware of these studies, it is now mandatory for our clinical trainees to take part in several courses on how to integrate and handle feedback and the CPSTs in clinical practice before seeing patients in the clinic. In addition, we offer ongoing support on how to use the system during treatment. This means that therapists with cases that have been identified by the TTN as being at risk for treatment failure are invited to take place in a feedback meeting. At this meeting, therapists can discuss the feedback system’s suggestions and reflect on what might be important for clinical practice to adapt treatment respectively.

The navigation system was evaluated in a prospective trial examining 377 patients. Compared to the usual care control group, a positive effect size difference of Cohen’s d = 0.2 was observed when therapists followed the recommended treatment strategy during the first eight sessions. Furthermore, the usage of psychometric feedback, therapist attitudes towards feedback, in-session use of feedback as well as self-reported confidence to use feedback were significant moderators (Lutz et al. submitted).

## Future Directions

In order to improve our measurements and therefore further improve the navigation system’s predictive power in the future, we have initiated several projects that utilize intensive measurements and technological advancements in video analyses. For example, we are currently using EMA in order to gather additional patient information several times a day. In this EMA project, we collect patient self-report data and also use smart watches that allow us to objectively measure biological parameters, such as heart rate variability, sleep duration, and activity. The aim is to deliver fine-grained feedback to both the therapist and patient and integrate reliable findings, into the navigation system (Lutz et al. 2018). Furthermore, we are conducting several projects focusing on nonverbal behavior and emotion recognition using video recordings of sessions. The aim is to create external and automatized measurements that can go beyond patient self-reports or instruments (Baur et al. 2020; Paulick et al. 2018). In addition, various data sources (e.g., patient vs. clinicians) can be integrated to gain a comprehensive picture of patient severity and symptom change over the course of treatment. In our research clinic, patients and therapists each fill out the same questionnaire after each session, enabling us to evaluate how consistent therapists’ and patients’ perspectives are, for example, with regard to the therapeutic alliance.

On the other hand, making use of recent technological advancements is not the only path improving the quality of our measures. In a further project, we have developed a video rating inventory capable of adequately assessing a wide range of therapeutic interventions and skills in personalized psychological treatments (Boyle et al. 2019). Allowing clinicians to watch and evaluate the therapeutic process as it unfolds may provide rich data not captured by self-reports.

## Conclusions

Developments in the field of evidence-based personalized treatments aim to change how psychotherapy is conducted. These developments confront some of the main limitations in mental health services very accurately identified and described in Dr. Bickman’s manuscript. However, these developments demand a clear orientation on patients’ characteristics and individual developments as well as a high level of therapist flexibility. Therefore, it is necessary that therapists be open to psychometric feedback that may present information contrary to their clinical intuition. Such systems must be thoughtfully implemented and integrated into clinical training.

At this point, we therefore want to emphasize that the aim of such precision mental health systems is not to dominate over clinicians, but to provide important information that should be used to question one’s professional work and reflect on other possibilities to enhance treatment outcome for a specific patient. For clinical practice, this implies the need for scientifically trained therapists that understand the meaning of statistical recommendations and are able to see them as an enrichment for their work. Thus, the success of these recommendation systems will also rely on the development of a new generation of clinicians that are willing and able to incorporate them in their clinical practice. For this purpose, it will be fundamental to integrate these systems into the early stages of their careers as part of clinical training.
