# Correction to: Working Towards the Development and Implementation of Precision Mental Healthcare: An Example

**DOI:** 10.1007/s10488-021-01149-z

**Published:** 2021-07-27

**Authors:** Wolfgang Lutz, Brian Schwartz, Juan Martín Gómez Penedo, Kaitlyn Boyle, Anne‑Katharina Deisenhofer

**Affiliations:** Department of Psychology, University of Trier, 54286 Trier, Germany

## Correction to: Administration and Policy in Mental Health and Mental Health Services Research (2020) 47:856–861 10.1007/s10488-020-01053-y

The article “Working Towards the Development and Implementation of Precision Mental Healthcare: An Example” written by “Wolfgang Lutz, Brian Schwartz, Juan Martin Gomez Penedo, Kaitlyn Boyle, Anne‑Katharina Deisenhofer”, was originally published Online First without Open Access. After publication in volume 47, issue 5, pages 856–861 the author decided to opt for Open Choice and to make the article an Open Access publication. Therefore, the copyright of the article has been changed to © The Authors 2021 and the article is forthwith distributed under the terms of the Creative Commons Attribution 4.0 International License, which permits use, sharing, adaptation, distribution and reproduction in any medium or format, as long as you give appropriate credit to the original author(s) and the source, provide a link to the Creative Commons licence, and indicate if changes were made. The images or other third party material in this article are included in the article’s Creative Commons licence, unless indicated otherwise in a credit line to the material. If material is not included in the article’s Creative Commons licence and your intended use is not permitted by statutory regulation or exceeds the permitted use, you will need to obtain permission directly from the copyright holder. To view a copy of this licence, visit https://creativecommons.org/licenses/by/4.0.

The original article has been corrected.

